# Imaging-Guided Algorithmic Management of Mandibular Condylar Fractures: A 13-Year Institutional Analysis of 495 Joints

**DOI:** 10.3390/cmtr19020028

**Published:** 2026-06-11

**Authors:** Sonal Anchlia, Hetal Amipara, Zibran Khan, Jigar Barasara, Jigar Dhuvad, Hrushikesh Gosai

**Affiliations:** Department of Oral and Maxillofacial Surgery, Government Dental College and Hospital, Ahmedabad 380016, India; sonal.anchlia@gmail.com (S.A.); jbarasara@gmail.com (J.B.); drjigardhuvad1981@gmail.com (J.D.); hrushikeshgosai@gmail.com (H.G.)

**Keywords:** mandibular condylar fracture, intracapsular fracture, ORIF, disc displacement, facial nerve injury

## Abstract

(1) Background: Mandibular condylar fractures continue to be a subject of debate, traditionally framed as a choice between open and conservative management. However, this binary approach fails to adequately account for fracture-level anatomy, Temporomandibular joint (TMJ) involvement, and functional outcomes. (2) Purpose: To present an imaging-guided, fracture-level-based algorithm that integrates radiologic evaluation, surgical approach selection, fixation biomechanics, and functional rehabilitation. (3) Review Strategy: This invited review combines current evidence with a 13-year institutional experience involving 495 joints. High-resolution Computed Tomography (CT) Imaging was used to assess fracture morphology, displacement, and ramal height, while Magnetic Resonance Imaging (MRI) was selectively employed in intracapsular fractures to evaluate disc–condyle relationships when intra-articular involvement was suspected. Management decisions, including surgical approach and fixation strategy, were guided by an institutional algorithm tailored to fracture characteristics. (4) Results: Implementation of this approach yielded consistent and predictable outcomes. Mouth opening improved from approximately 18.77 mm preoperatively to 40 mm at 6 months. Lateral excursions became symmetrical (~9.6 mm), occlusion was restored in all patients, and bite force returned to near-physiological levels. Pain scores showed near complete resolution within 1 month. Postoperative morbidity remained low, with predominantly transient facial nerve weakness. (5) Conclusions: This imaging-guided, algorithmic framework provides reproducible functional outcomes and signifies a shift toward structured, anatomically driven management of condylar fractures.

## 1. Introduction

Mandibular condylar fractures account for approximately 25–45% of all mandibular fractures and remain among the most debated injuries in maxillofacial trauma [1,2]. For more than half a century, management strategies have oscillated between conservative functional treatment and surgical reconstruction, yet consensus regarding optimal treatment selection remains elusive [3,4]. The historical preference for conservative management was largely based on the adaptive capacity of the temporomandibular joint and concerns regarding facial nerve injury associated with surgical exposure [5,6]. Functional remodelling, neuromuscular adaptation, and occlusal compensation were therefore considered adequate in many fracture patterns [3]. However, advances in high-resolution imaging and long-term functional outcome assessment have demonstrated that this approach is not universally applicable, particularly in displaced and functionally disruptive fracture configurations [7]. Loss of ramal height, angular deformity, persistent deviation, malocclusion, and temporomandibular joint dysfunction remain well-recognized sequelae when anatomical reduction is not achieved in appropriately selected cases [6,7].

Despite the substantial expansion of clinical literature over recent decades, clarity in decision-making remains limited [8]. Importantly, this persistent controversy does not reflect a lack of evidence, but rather fragmentation of evidence across multiple domains. Imaging criteria, fracture classification systems, operative indications, surgical approach selection, fixation biomechanics, and intra-articular soft-tissue considerations are frequently evaluated in isolation rather than integrated into a unified treatment strategy. As a result, classification systems describe anatomy but rarely guide management, and operative thresholds continue to vary between surgeons and institutions.

This challenge is particularly evident in intracapsular injuries, where disruption involves not only the osseous components but also the disc-capsule-vascular complex of the temporomandibular joint [9]. Similarly, surgical approach selection and fixation strategies are often determined by surgeon preference rather than fracture morphology and biomechanical requirements. Consequently, the central question in condylar fracture management is no longer whether open or conservative treatment is superior in absolute terms, but rather which fracture, in which patient, at which time point, requires which intervention, through which surgical approach, and with which fixation strategy to optimize long-term functional outcomes while minimizing morbidity.

**Figure 1:**
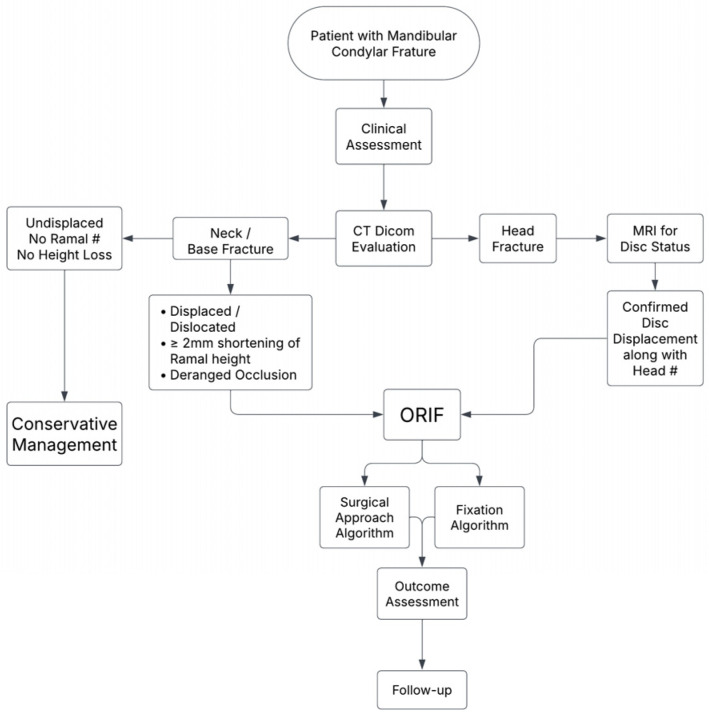
Decision-making Algorithm.

**Figure 2:**
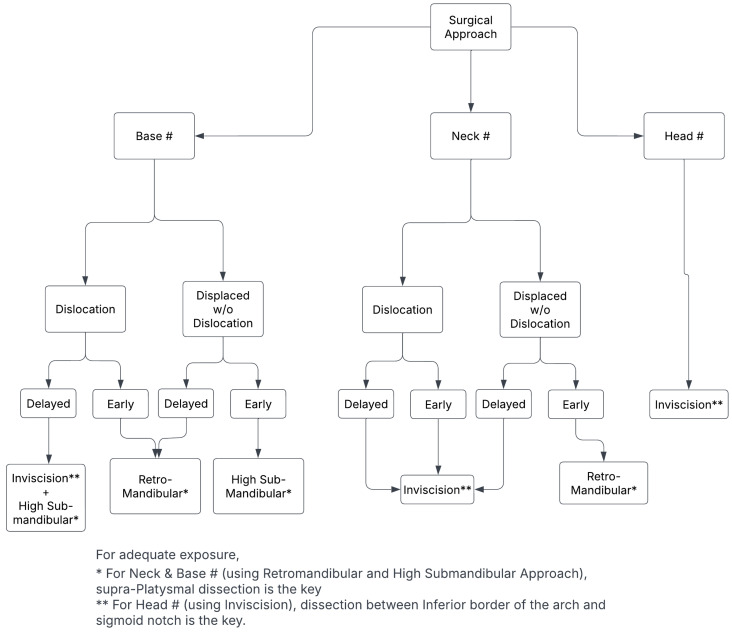
Surgical Approach Selection Algorithm.

**Figure 3:**
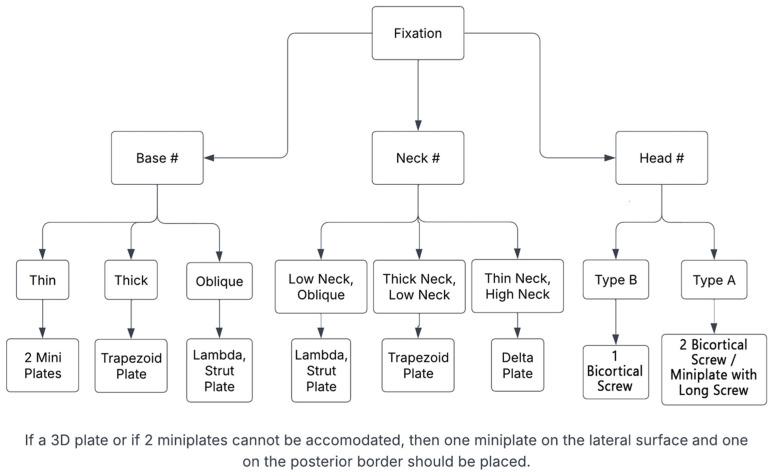
Fixation Selection Algorithm.

In this review, we propose an imaging-guided algorithm that integrates radiologic morphology, disc position, fracture level, surgical access strategy, fixation biomechanics, and rehabilitation into a unified treatment pathway (Figure 1, Figure 2 and Figure 3) supported by outcomes from 495 surgically man-aged condylar fractures treated over 13 years [10]. By integrating radiologic parameters, fracture morphology, temporomandibular joint disc status, surgical access strategy, fixation biomechanics, and functional rehabilitation into a single reproducible decision-making pathway, this work aims to transition condylar fracture management from a historically controversial topic to a structured and predictable algorithmic discipline.

## 2. Evaluation of Treatment Philosophy

### 2.1. Principle 1: Radiological Evaluation as the Foundation of Decision-Making

Treatment planning for mandibular condylar fractures must be based on high-resolution Computed Tomography (CT) with direct DICOM evaluation rather than conventional radiographs (OPG, PA, AP), which are limited by distortion and superimposition. Although 3D reconstructions are useful for diagnosing fracture level, they can mask subtle fracture lines, rotational deformities, and medial displacement due to thresholding limitations. Additionally, preformatted CT sections may not accurately depict the actual direction or extent of displacement; therefore, the surgeon must systematically analyze the entire multiplanar DICOM. Sagittal sections best reveal anterior displacement, coronal sections identify medial displacement, and axial sections assess rotational alignment and adequacy of reduction. This systematic multiplanar interpretation forms the foundation of the proposed algorithmic decision-making pathway.

### 2.2. Principle 2: Reinterpreting the Indications of Open vs. Closed Management

The classic indications outlined by Zide and Kent (1983) remain among the most frequently cited frameworks guiding surgical decision-making in mandibular condylar fractures and continue to be deeply embedded in surgical teaching and literature [3]. Their seminal work established absolute indications for open reduction—including displacement of the condyle into the middle cranial fossa, the presence of a foreign body, lateral extracapsular displacement, and the inability to achieve satisfactory occlusion—alongside relative indications applicable primarily to adult patients with displaced condyles and associated malocclusion [3]. Around the same time, Mathes and Hentz (1983) proposed additional criteria emphasizing biomechanical and morphological indicators of instability, including fragment angulation greater than 30°, loss of bony contact exceeding 4–5 mm, and persistent malocclusion after attempted conservative management (Table 1) [4]. These observations expanded the discussion from simply when surgery should be performed to why operative intervention may be necessary based on structural determinants of dysfunction. However, despite recognizing these instability patterns, their recommendations did not gain widespread acceptance, likely reflecting the dominant treatment philosophy of that period, when conservative management with MaxilloMandibular Fixation (MMF) was considered the standard approach. Concerns regarding facial nerve injury, limited surgical exposure, and the relatively primitive osteosynthesis systems available at the time further reinforced the preference for conservative management.

Over the past two decades, the management paradigm of mandibular condylar fractures has evolved substantially [7]. Landmark prospective randomized multicenter studies by Eckelt et al. (2006) and Schneider et al. (2008) provided level I evidence demonstrating that open reduction and internal fixation (ORIF) yields significantly superior outcomes compared to conservative management in displaced condylar fractures, with better mouth opening, fewer occlusal disturbances, and lower pain scores [11,12]. Eckelt et al. reported a mean interincisal distance of 46.5 mm in the operatively treated group versus 40.9 mm in the conservatively managed group (*p* = 0.01), while Schneider et al. confirmed a 12 mm advantage in mouth opening with ORIF across all fracture levels [11,12]. These findings, irrespective of fracture level, provided a strong scientific basis for expanding operative indications. Contemporary decision-making increasingly relies on CT-based assessment of fracture level, displacement, and ramal height loss, allowing more precise characterization of fracture morphology and its functional implications. These principles are consistent with the practice parameters of the American Association of Oral and Maxillofacial Surgeons (AAOMS), which emphasize restoration of stable occlusion, mandibular function, and posterior facial height as primary therapeutic objectives, thereby facilitating more rational identification of patients who may benefit from open reduction and internal fixation. Importantly, AAOMS guidelines also recognize the inability to tolerate prolonged MMF as a relative indication for operative management, implicitly acknowledging the significant functional burden associated with extended mandibular immobilization [13].

Yet an important dimension remains insufficiently integrated into most contemporary decision frameworks: patient-centered functional recovery. Prolonged immobilization restricts mastication, speech, oral hygiene, and social interaction, thereby negatively affecting early recovery and overall quality of life [13]. Despite these well-recognized consequences, contemporary protocols continue to prioritize radiographic displacement and occlusal parameters while largely overlooking the patient-experienced impact of extended immobilization. This represents a critical gap in current treatment protocols and underscores the need for more patient-centered decision frameworks in condylar fracture management.

These observations are further supported by recent evidence. A comprehensive systematic review published in 2025 by Youssef et al. identified 121 distinct indications across 100 studies, highlighting the considerable variability that continues to characterize treatment decision-making [8]. Notably, most reported indications ultimately trace back to the classic criteria proposed by Zide and Kent, despite being supported largely by low-level or opinion-based evidence. Commonly cited displacement thresholds, such as angulation ≥10° or ramal height shortening ≥2 mm, therefore remain largely empirical rather than biologically validated. Collectively, these findings emphasize the persistent uncertainty surrounding operative indications and reinforce the need for more objective, imaging-guided, and patient-centered frameworks for decision-making in mandibular condylar fracture management [8].

### 2.3. Indication of Intracapsular Condylar Fracture and the Role of Disc Position

The controversy surrounding management is particularly pronounced in intracapsular (condylar head) fractures, which represent true intra-articular injuries of the temporomandibular joint [14]. From a broader musculoskeletal perspective, orthopaedic trauma principles emphasize that displaced intra-articular fractures require anatomical reduction to restore joint congruity and prevent long-term degenerative sequelae [15]. A similar rationale applies to intracapsular fractures of the mandibular condyle, where disruption involves not only the articular surface but also the disc–condyle–capsular complex, with potential impairment of temporomandibular joint biomechanics [14].

Unlike primary internal derangement, in which the osseous framework remains intact and adaptive remodelling may occur, condylar head fractures frequently involve displacement of the articular disc together with the fractured condylar fragment, resulting in loss of the structural support necessary for normal joint adaptation. In such situations, reliance on spontaneous remodelling may be overly optimistic, and persistent intra-articular instability may compromise long-term joint function. Importantly, even when occlusion appears acceptable, significant intra-articular damage may remain unrecognized, increasing the risk of temporomandibular joint dysfunction or ankylosis in both children and adults.

**Figure 4:**
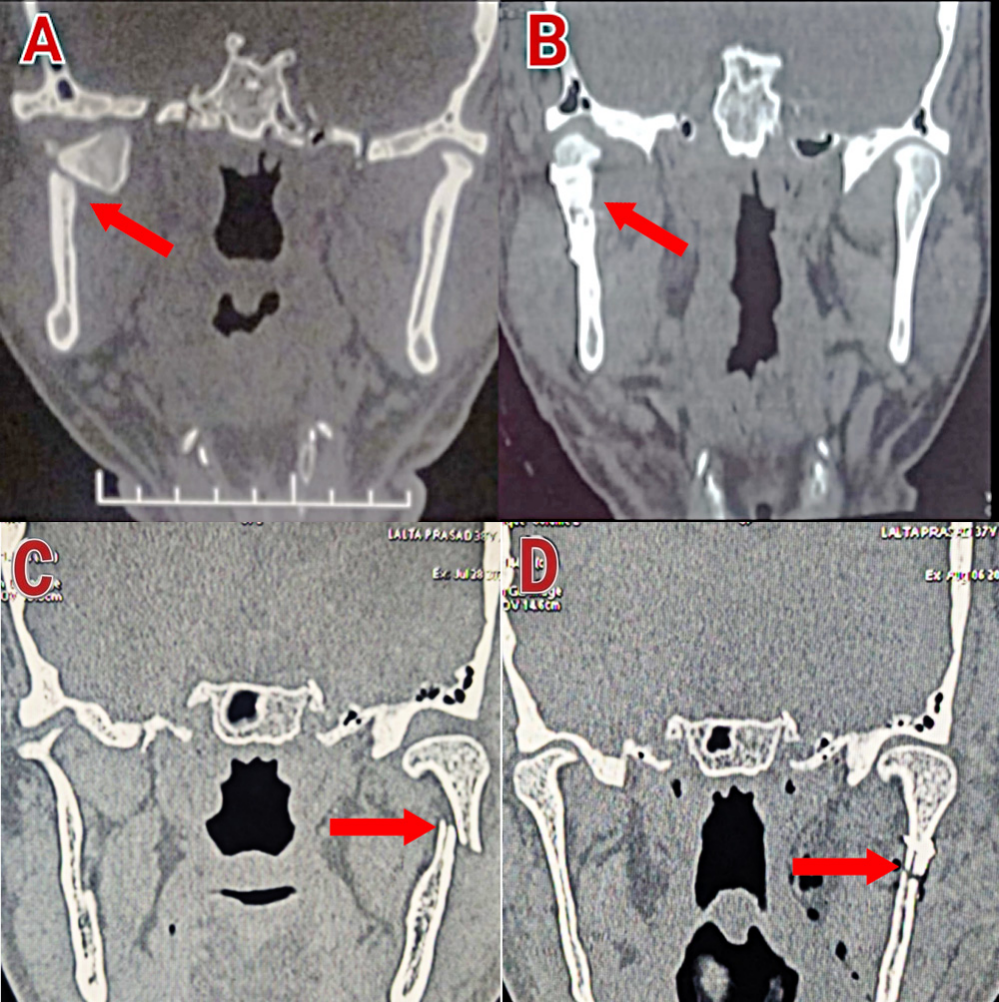
CT Image showing Pre-operative and Post-operative Reduction. (**A**) Pre-operative CT image of Condylar Base Fracture. (**B**) Post-operative CT image of Condylar Base Fracture. (**C**) Pre-operative CT image of Condylar Neck Fracture. (**D**) Post-operative CT image of Condylar Neck Fracture.

**Figure 5:**
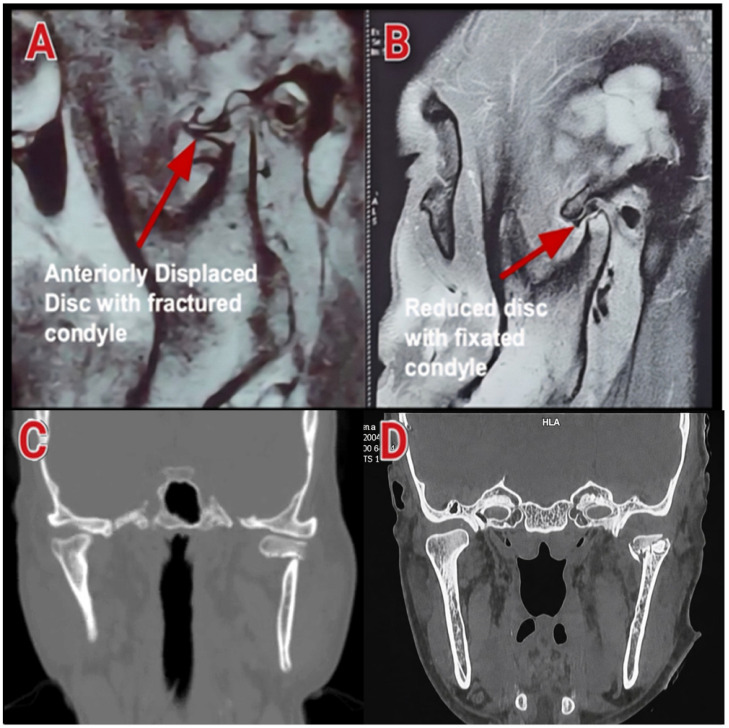
CT & MRI Image showing Pre-operative and Post-operative Reduction. (**A**) Pre-operative MRI image showing an Anteriorly Displaced Disc. (**B**) Post-operative MRI image showing Normally Positioned Disc. (**C**) Pre-operative CT image of Condylar Head Fracture. (**D**) Post-operative CT image of Condylar Head Fracture.

Several referral patients with displaced intracapsular fractures initially treated conservatively elsewhere later presented with temporomandibular joint ankylosis (15 cases) or internal derangement (55 cases), underscoring the limitations of relying solely on occlusion to determine injury severity in intracapsular fractures. In this context, using Magnetic Resonance Imaging (MRI) for detection of intra-articular derangement provides valuable additional information and assists in identifying patients who may benefit from open joint surgery aimed at restoring anatomical alignment and preserving long-term temporomandibular joint function (Figure 4 and Figure 5).

He et al. classified intracapsular condylar fractures into distinct subtypes based on fracture morphology and displacement pattern [9,14]. In this classification, Type A fractures involve a single fracture line with a larger proximal fragment retaining a degree of structural integrity, while Type B fractures involve a smaller or more comminuted proximal fragment with greater displacement and reduced bone stock [9]. He et al. support open reduction for intracapsular fractures, particularly Types A and B, due to their increased risk of temporomandibular joint dysfunction. This approach becomes especially important in the presence of ramal shortening and when the ramal stump comes into contact with the zygomatic arch, as these factors may elevate the risk of ankylosis [14]. Moreover, arthroscopic findings at our institute also reveal concomitant intra-articular injury, indicative of potential internal derangement, thereby further justifying surgical intervention in intracapsular fractures.

### 2.4. Principle 3: Surgical Approach Selection Based on Fracture Level

Selection of surgical access in mandibular condylar fractures should be determined primarily by fracture level, displacement pattern, and fixation requirements rather than surgeon preference alone. Historically, reluctance to perform open reduction in condylar fractures was largely influenced by concerns regarding facial nerve injury and limited surgical exposure [5,14,16]. However, refinement of surgical approaches and improved understanding of regional anatomy have significantly reduced approach-related morbidity, allowing more predictable access to different fracture levels of the condyle.

### 2.5. Approach Selection According to Fracture Level

Because the condylar head, neck, and base represent anatomically distinct regions with different accessibility and fixation constraints, surgical exposure should be tailored accordingly.

For mandibular condylar head fractures, a Peri meatal modification of the Inviscision approach, recently described at our institute, represents a cosmetically superior alternative that provides direct and adequate access for precise osteosynthesis [17,18]. Soft tissue is reflected along the zygomatic arch, between the sigmoid notch and the inferior border of the arch, facilitating optimal visualization of the condylar head. Reduction is further facilitated using a screw-wire traction technique applied to the mandibular ramus, enabling controlled inferior distraction and accurate repositioning of dislocated condylar fragments—a manoeuvre originally described by Ellis III in his Atlas of Approaches to the Craniofacial Skeleton, in which wire is applied to the distal segment to achieve controlled inferior traction and facilitate retrieval of the displaced proximal fragment [19]. Notably, approximately 80% of condylar head fractures in our series demonstrated displacement, predominantly attributable to the anteromedial pull of the lateral pterygoid muscle on the proximal fragment. Postoperative transient facial nerve weakness was observed secondary to soft tissue retraction; however, all cases demonstrated complete functional recovery within 3–6 months. The incidence of facial nerve morbidity with this approach appears comparatively low when contrasted with existing literature. A systematic review by Al-Moraissi et al. (2018) reported transient facial nerve weakness rates of 8.5–11.5% for the preauricular approach and approximately 0–1% for the deep subfascial modification [5].

For condylar neck fractures, the retromandibular transparotid approach offers a short working distance and excellent fracture visualization. However, intraparotid dissection increases the risk of facial nerve injury and salivary complications [14]. Reported rates include 14–19% transient facial nerve weakness and ~1.4% permanent injury (Al-Moraissi et al., 2018) [5]. To mitigate these risks, a modified approach—the retromandibular sub-parotid approach—has been advocated, in which the parotid gland is retracted superiorly without intraglandular dissection, thereby reducing the likelihood of facial nerve injury and postoperative salivary fistula (Scolozzi & Foletti, 2020) [20]. In both the retromandibular sub-parotid and the high submandibular approaches, the facial nerve is routinely identified, dissected, and skeletonised under direct vision. This deliberate exposure provides adequate scope for careful retraction without traction injury, and accounts for the absence of any permanent facial nerve injury in our series using these two techniques. In our experience, no facial nerve weakness was observed with retromandibular transparotid approach. Four early cases developed parotid fistula; after modification of the SMAS-parotid fascia-platysma closure technique, no further fistula occurred.

For condylar base fractures, the high submandibular approach described by many authors [18,19,21,22] provides direct access to the posterior border of the ramus while remaining inferior to the major facial nerve branches, resulting in very low facial nerve injury rates (0–6%) (Al-Moraissi et al., 2018) [5]. Additionally, the resulting scar is well concealed within the shadow of the inferior border of the mandible, offering a favourable cosmetic outcome. In contrast, the traditional low submandibular (Risdon) approach, which requires deeper dissection beneath the marginal mandibular branch, has been associated with higher rates of transient facial nerve weakness (~15%) (Al-Moraissi et al., 2018) [5]. No facial nerve weakness was observed with this approach.

### 2.6. Principle 4: Sequence of Reduction in Combined Fracture

The ideal sequence of fixation in mandibular fractures affecting both the condylar region and the tooth-bearing part of the mandible remains a subject of debate. Traditionally, fixation of the anterior mandibular fracture was recommended first on the basis that restoring the dental arch and occlusion creates a stable reference point for subsequent reduction in the condylar segment. Ellis and Throckmorton highlighted that stabilizing the tooth-bearing mandible aids in re-establishing occlusion under maxillomandibular fixation [23], while Champy and colleagues similarly advocated for restoring mandibular arch continuity before addressing associated condylar fractures [24].

However, this concept has been increasingly questioned, particularly in cases with significant condylar displacement or loss of ramal height. Zide and Kent, Neff et al., and Al-Moraissi et al. [2,3,7] emphasized that precise anatomical reduction in the condyle is critical for restoring occlusion, mandibular biomechanics, and temporomandibular joint function. Kamath et al. also demonstrated that early reduction in the condylar segment restores ramal and posterior facial height, establishing a stable anatomical reference for subsequent fixation of the anterior mandible; an easy-to-pull distal segment and minor anterior discrepancies (1–2 mm) can be compensated at the anterior mandible [25]. This strategy is applicable primarily in acute cases; in delayed presentations, anterior segment fixation should be performed first, as occlusion serves as the primary guide for precise reduction.

Based on these considerations, our protocol adopts a condyle-first strategy in combined fractures involving the condyle and anterior mandible and has achieved successful results. In this approach, the condylar fracture is reduced and stabilized first to restore ramal height and anatomical seating within the glenoid fossa, after which the tooth-bearing segment is reduced and fixed under controlled occlusion.

### 2.7. Principle 5: Fixation Biomechanics

The selection of fixation strategy in mandibular condylar fractures should be guided primarily by fracture level, fragment size, available bone stock, and the biomechanical loading pattern across the condylar region. Condylar fractures are subjected to complex rotational and translational forces generated by the lateral pterygoid muscle and functional mandibular movements, making stable three-dimensional fixation essential for maintaining reduction during early mobilization.

For condylar head fractures, fixation is frequently limited by the small size of the proximal fragment and restricted bone stock, which may preclude placement of multiple plates. Consequently, fixation is commonly achieved using positional bicortical screws or small microplates, which provide stable fixation while minimizing hardware bulk within the temporomandibular joint region. As clarified by Eckelt et al., stable fixation in the condylar head is best achieved using positional bicortical screws rather than a true lag screw construct, given the limited bone volume and strength available in this region [11]. Ellis et al. and He et al. reported that screw fixation in condylar head fractures provides stable reduction with satisfactory functional outcomes while preserving joint integrity [14]. In accordance with He’s classification, we applied two positional bicortical screws for Type A fractures, which have a larger proximal fragment and sufficient bone stock to accommodate dual-screw fixation, and a single positional bicortical screw for Type B fractures, where the smaller or more comminuted fragment limits the placement of a second screw [9,14]. When additional stability was required, a miniplate was combined with a long screw (12–14 mm). Importantly, all fixations should be placed away from the articulating surface.

For condylar neck and base fractures, fixation is traditionally performed using two miniplates in a triangular configuration, based on Meyers’ tension lines [26]. One plate is placed along the posterior border of the ramus to resist compressive forces, and the other below the sigmoid notch to counter tensile forces, providing stable three-dimensional fixation [27]. This technique, popularized by Ellis and Dean, has also been supported by Meyer et al., who further supported double-plate fixation, reporting reliable stability and low hardware failure [1,2]. Placement of two miniplates in the condylar region is often challenging due to limited bone surface and restricted access. Therefore, three-dimensional (3D) plates have been introduced to achieve stable fixation with a single construct. Recent systematic reviews also indicate that 3D plates provide reliable fixation with shorter operative time and fewer hardware complications, while maintaining comparable functional outcomes (Al-Moraissi et al.) [28]. The delta plate is widely used for condylar neck fractures; Ahuja et al. demonstrated favorable outcomes compared with conventional miniplates, particularly in thin condylar neck fractures [29], while finite element studies show improved stress distribution with triangular plate designs (Murakami et al.) [30]. The lambda plate also provides triangular fixation with multiple screw options and has been associated with adequate stability and reduced operative time (Meyer et al.; Neff et al.), although adaptation may be difficult in narrow condylar necks, and screw crowding in the proximal fragment can limit optimal placement [2,26]. For condylar base fractures, broader plates such as the trapezoid plate have been advocated. This design offers wider fracture coverage and improved screw distribution, providing stable fixation while reducing the need for multiple plates (Choi et al.; Alkan et al.) [31,32].

### 2.8. Principle 6: Functional Rehabilitation as an Integrated Component

A notable limitation of prior comparative studies is the isolation of surgical intervention from postoperative rehabilitation. Condylar fractures are dynamic injuries affecting neuromuscular coordination and joint function. In our series, mouth opening exercises were commenced on the first day after surgery in all patients following stable fixation. Early guided mandibular mobilization was initiated within the first postoperative week in most patients, except for 2 cases. Patients were instructed to maintain a soft diet postoperatively and to avoid hard food until adequate healing was confirmed clinically and radiologically. Owing to stable rigid fixation and precise occlusal restoration, postoperative elastic guidance was not required, underscoring the importance of integrating surgical accuracy with functional rehabilitation.

## 3. Institutional Experience Supporting the Algorithm

### 3.1. Cohort Description

This invited review integrates current evidence with retrospective analysis of a prospectively maintained institutional surgical database over 13 years (2013–2026) at a tertiary referral center. This cohort reflects surgically managed condylar fractures selected according to institutional algorithmic criteria rather than the full spectrum of condylar fracture presentations. It reflects a continuously evolving, experience-driven surgical practice, allowing longitudinal evaluation of treatment strategies and outcomes across varying fracture patterns and clinical scenarios.

### 3.2. Cohort Eligibility

Patients included in this clinical cohort met the following criteria:

1. Age ≥16 years
2. Functional impairment was defined as deranged occlusion, restricted mouth opening, or clinically evident mandibular deviation during movement
3. Radiologic evidence of displacement ≥10° or ramal height shortening ≥2 mm
4. Fracture dislocation
5. Contraindication to intermaxillary fixation
6. MRI-confirmed disc displacement, even in the absence of occlusal derangement

Patients were excluded if they had:

1. Edentulous status
2. Pathological fractures
3. Pre-existing temporomandibular joint internal derangement unrelated to trauma
4. Associated midfacial fractures
5. Medical contraindications to surgical intervention

Six-month follow-up data were available for all patients included in the functional outcome analysis. Fractures were classified according to Loukota et al. Patients were allocated to one of three surgical approaches based on fracture level, dislocation, and timing post-injury (Surgical approach algorithm)

**Figure 6:**
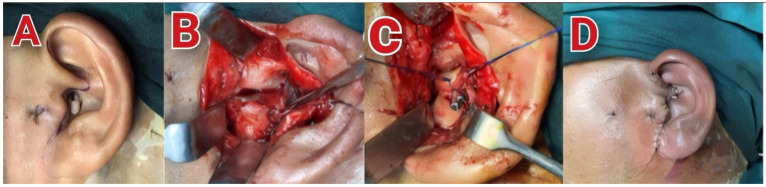
Inviscision Approach. (**A**): Marking of the incision; (**B**): Exposure of Fracture; (**C**): Fixation of Condylar Head with 2 Long Screw; (**D**): Closure of Incision.

**Figure 7:**
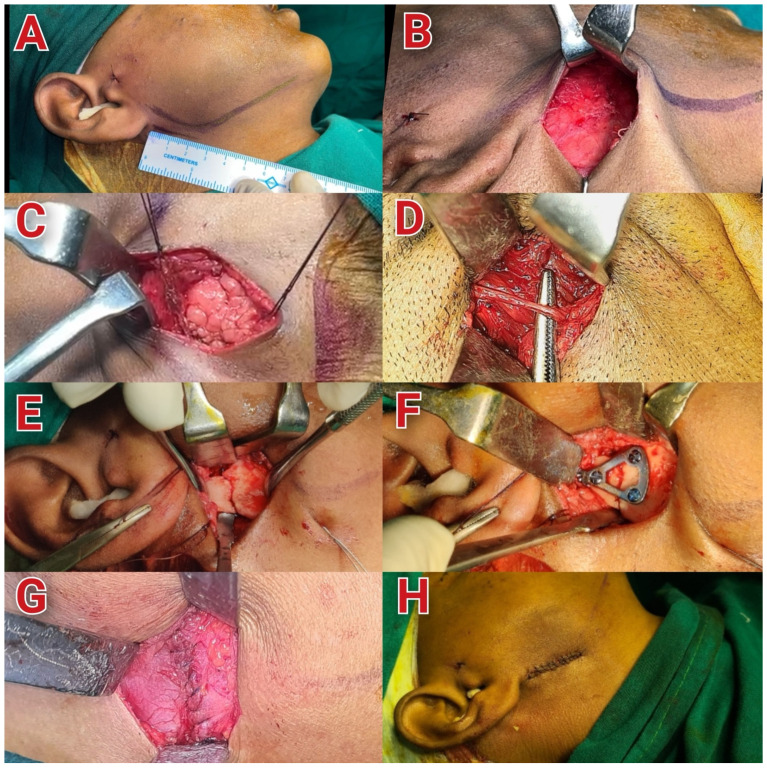
Retromandibular Approach. (**A**) Marking of the incision. (**B**) Supra-platysmal Dissection. (**C**) SMAS, Platysma, and parotid capsule Cut together. (**D**) Buccal Branch of the facial nerve. (**E**) Exposure of Fracture Fragment. (**F**) Fixation of Condylar Neck with Delta Plate. (**G**) SMAS, Platysma, and parotid capsule suture together. (**H**) Closure of Incision.

**Figure 8:**
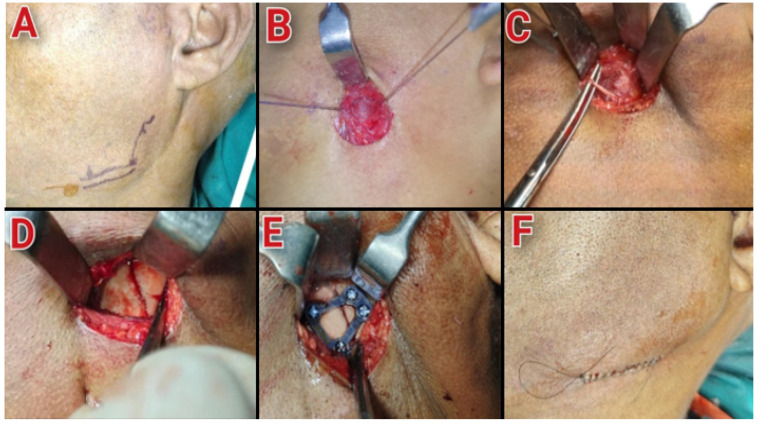
High Submandibular Approach. (**A**) Marking of the incision. (**B**) Vertical Cut in Platysma; (**C**) Buccal Branch of the facial Nerve. (**D**) Exposure of Fracture Fragment. (**E**) Fixation of Condylar Base with Trapezoid Plate. (**F**) Closure of Incision.

- Group A: Intracapsular fracture (Inviscision (Figure 6) & Endaural)
- Group B: Condylar neck fracture (Retromandibular-subparotid) (Figure 7)
- Group C: Condylar base fracture (High submandibular) (Figure 8)

For dislocated fractures, a higher-level approach was used. Delayed, malunited, or complex fractures were managed using an Invicision approach, with an additional high submandibular approach when required.

### 3.3. Surgical Technique

The surgical techniques described below reflect the standardized institutional protocol supporting the proposed treatment algorithm (Figure 2). All operations were performed under general anesthesia with nasotracheal intubation. Standardized incisions (Inviscision, retromandibular-sub parotid, or high submandibular) were employed. Facial nerve branches were identified, dissected, and skeletonised under direct vision in all cases using the retromandibular sub-parotid and high submandibular approaches, providing adequate scope for careful retraction and ensuring preservation of neural integrity. In markedly displaced fractures, the surgical approach was escalated one level proximally to facilitate reduction using the retromandibular-subparotid approach for condylar base fractures and the Inviscision approach for condylar neck fractures. In delayed presentations, an Inviscision approach was routinely combined with high submandibular, even for neck and base fractures, to allow adequate exposure of the temporomandibular joint and facilitate removal of fibrous tissue from the glenoid fossa, thereby enabling proper reduction in the condylar segment. Subperiosteal dissection was performed to expose the fracture site. Fracture reduction was performed using the screw-wire technique, as originally described by Ellis III, facilitating controlled inferior traction of the ramus and retrieval of the displaced proximal fragment with a specialized indigenous instrument [19].

Fixation method (Figure 3): Condylar neck and base fractures: 2.0 mm 3D delta/trapezoid or 2 mm mini plates with 2.0 × 6 mm titanium screws.

- Condylar head fractures: positional bicortical screws or small microplates, depending on fragment size and morphology, placed away from the articulating surface.

In cases with disc displacement, the articular disc was repositioned to its anatomical position and rigidly anchored using non-resorbable sutures secured to the fixation hardware (Figure 6). When the disc was irreparably torn, discectomy was performed, followed by interpositional placement of an abdominal dermal fat graft harvested through a periumbilical incision.

### 3.4. Functional Outcomes Assessment

The cohort included 413 patients with 495 condylar fractures, including 82 bilateral injuries. All demographic details are outlined in Table 2. Statistical analysis was performed using repeated-measures ANOVA with significance set at *p* < 0.05. Clinical outcomes showed strong and statistically significant functional recovery (Table 3). Types of fixation are detailed in Table 4. Functional improvement was progressive and statistically significant across all postoperative time intervals, demonstrating early recovery within the first postoperative week and near-complete restoration of mandibular function by 6 months. Maximal mouth opening significantly increased from a preoperative mean of 18.96 ± 1.98 mm to 26.21 ± 1.92 mm at 1 week, 35.47 ± 1.91 mm at 1 month, and 40.53 ± 2.73 mm at 6 months (*p* < 0.001), indicating excellent functional recovery. Lateral excursions also improved symmetrically, reaching approximately 9.6 mm bilaterally at 6 months (*p* < 0.001). Pain-Free Maximal Bite Force was measured using a dedicated bite force measurement device in which the patient bites onto calibrated blades; the resultant force is displayed digitally in Newton (N) units. The protocol was strictly aligned with the fourth AO Principle of Internal Fixation, which advocates for ‘early, active pain-free mobilization’ [33]. Bite force analysis at 6 months revealed effective masticatory restoration, with anterior bite force of 120.40 ± 7.40 N and posterior forces of 343.78 ± 19.52 N (left) and 339.43 ± 23.37 N (right). Occlusion normalized in 99% of patients within 1 week and in all cases by 6 months (*p* < 0.001). Pain scores (VAS) decreased markedly from 7.88 ± 0.61 preoperatively to 2.95 ± 0.53 at 1 week, with near-complete resolution by 1 month and complete remission by 6 months (*p* < 0.001). (Table 3)

Postoperative morbidity remained low. Parotid fistula was observed in 4 cases, while facial nerve injury occurred in 10% of patients. Notably, the majority of nerve injuries were transient and resolved during follow-up, with only a single case (0.2%) resulting in a permanent deficit. Extracorporeal fixation was required in 2 cases. Additionally, 2 patients with condylar head fractures required reoperation due to the use of excessively long screws (Table 2).

## 4. Limitations

This work represents an invited review that draws on institutional experience and available evidence to develop an imaging-guided, algorithmic treatment framework. As with any such effort, certain limitations warrant consideration. The underlying data is primarily retrospective and derived from a single institution, which introduces the possibility of selection bias and restricts how broadly the findings can be applied. Because patients were not randomized, the ability to draw firm causal conclusions is limited. Additionally, while objective clinical and radiologic outcomes are well documented, the study gives relatively little attention to patient-reported outcomes and quality-of-life measures. Prospective, multicenter investigations that incorporate validated functional scoring tools and patient-centered endpoints will be important for confirming and extending the generalizability of this algorithmic approach.

## 5. Conclusions

Management of mandibular condylar fractures should move beyond the traditional open-versus-closed dichotomy toward an imaging-guided approach integrating fracture level, displacement, disc position, biomechanical stability, and functional goals. CT defines fracture morphology and instability, while MRI provides important decision-modifying information in intracapsular injuries. Fracture-specific surgical approaches, appropriate fixation, and early rehabilitation together enable reliable restoration of ramal height, occlusion, and mandibular mobility. Our experience with 495 joints over 13 years demonstrates that this algorithmic strategy is reproducible and yields predictable outcomes. This represents a paradigm shift from controversy-driven management to a standardized, anatomically driven framework for modern condylar fracture care.

## Figures and Tables

**Table 1:** Multi-System Comparison of Treatment Indications for Mandibular Condylar Fractures.

Criteria	Zide and Kent	Mathes	AAOMS/AOMSI	AO CMF	Institutional Algorithm (Present Study)
Absolute indications	Cranial fossa displacement; foreign body; lateral extracapsular displacement; inability to achieve occlusion	Dislocated condyle; loss of fragment contact	Failure of conservative management; mechanical obstruction; foreign body	Severe displacement with functional block	Mechanical obstruction to mouth opening; TMJ dysfunction due to dislocation
Displacement/Angulation	Lateral override	>30° angulation	Significant displacement	>30–45° angulation	>10° proximal segment angulation
Ramal height loss	Indirect	Ramus shortening (~≥5%)	Considered if occlusion affected	>2–5 mm shortening	>2 mm ramal shortening
Occlusion	Inability to achieve occlusion	Malocclusion after conservative management	Persistent malocclusion	Primary determinant	Included (functional derangement)
Functional limitation	Limited movement	Functional impairment	Mandibular dysfunction	Mouth opening restriction	Mechanical obstruction to opening
Dislocation	Lateral extracapsular displacement	Condyle out of fossa	Dislocation present	Strong indication	Dislocation causing TMJ dysfunction
Fragment relation	Not defined	Loss of fragment contact; bone gap >4–5 mm	Not explicit	Loss of alignment	Superolateral override (zygomatic arch impingement)
TMJ/Soft tissue	Not included	Dysfunction risk	Hemarthrosis; effusion; CSF otorrhea	Ankylosis risk	MRI-confirmed disc tear/TMJ dysfunction
Bilateral fractures	Relative indication	Bilateral with open bite	Bilateral with open bite	Functional deficit important	Considered selectively
Conservative indications (CM)	Not defined	Not defined	Undisplaced fractures	Functional stability	Undisplaced; comminuted; pediatric without displacement

**Table 2:** Demographic parameters.

Parameter	Sub-Category	Value (*n* %)
**Demographics**		
Age (years)	Mean ± SD (Range)	31.03 ± 12.64 (18–65)
Gender	Male	329 (79.7%)
	Female	84 (20.3%)
	**Total Patients**	413
**Fracture Characteristics**		
Associated Fracture	Parasymphysis	178 (43.1%)
	Body	92 (22.3%)
	Angle	61 (14.8%)
	Symphysis	48 (11.6%)
	Dentoalveolar	8 (1.9%)
	Absent	26 (6.3%)
Condyle Fracture Distribution	Base	224 (45.25%)
	Head	206 (41.62%)
	Neck	65 (13.13%)
	**Total Joints**	495
**Operative Parameters**		
Days from trauma to surgery	Mean ± SD (Days)	9.71 ± 4.03
Incision to exposure time	Mean ± SD (min)	9.59 ± 2.78
Fixation time	Mean ± SD (min)	8.74 ± 2.71
**Postoperative Complications**	Parotid Fistula	4 (1.0%)
	Facial Nerve Injury (Total)	41 (10.2%)
	Temporary Injury	40 (10%)
	Permanent Injury	1 (0.2%)

**Table 3:** Functional Outcome Parameters Over Time.

Time Interval	Mouth Opening (mm) (Mean ± SD)	Normal Occlusion (% Patients)	Left Posterior Bite Force (N) (Mean ± SD)	Right Posterior Bite Force (N) (Mean ± SD)	Right Excursion (mm) (Mean ± SD)	Left Excursion (mm) (Mean ± SD)	VAS Score (Mean ± SD)
Pre-operative	18.77 ± 2.30	Deranged (100%)	55.00 ± 15.00	50.00 ± 14.00	5.27 ± 4.04	5.13 ± 3.15	7.90 ± 0.74
1 Week	25.81 ± 1.91	Normal (99.0%)	110.00 ± 20.00	105.00 ± 19.00	6.79 ± 2.45	5.94 ± 2.33	2.90 ± 0.54
1 Month	35.32 ± 2.02	Normal (99.0%)	274.47 ± 20.00	270.00 ± 22.00	8.69 ± 1.33	8.25 ± 1.60	0.06 ± 0.24
6 Months	40.55 ± 1.88	Normal (100%)	343.78 ± 19.52	339.43 ± 23.37	9.56 ± 0.62	9.63 ± 0.77	0.00 ± 0.00
*p* value	<0.001	<0.001	<0.001	<0.001	<0.001	<0.001	<0.001

**Table 4:** Distribution of Fixation Methods according to Fracture Level.

Fracture Site	N = 495	Fixation Method	N	Percentage
Condylar Base	224	Trapezoid Plate	146	65%
		Two Miniplates	78	35%
Condylar Neck	65	Delta Plate	49	76%
		One Miniplate	16	24%
Condylar Head	206	Double Bicortical Screws	103	50%
		Single Bicortical Screw	62	30%
		Miniplate	41	20%
Total	495		495	100%

## Data Availability

The data presented in this study are available on request from the corresponding author due to privacy and ethical restrictions.
